# Abscess

**Published:** 1862-02

**Authors:** 


### ABSCESS.

In the editorial columns of the Register of May last we gave a case of alveolar abscess, its condition, treatment, and the result up to that time; shortly after that we lost sight of it. We have this day, Feb. 6th, examined the case and find the parts in perfect health, which have been so since we last saw the case, except that upon taking cold there would be a slight irritation, which would with the proper precaution soon pass away. The general health is much improved, and the teeth and mouth are in a healthy condition; and without doubt with the proper care will remain so. T.

